# XELIRI-bevacizumab versus FOLFIRI-bevacizumab as first-line treatment in patients with metastatic colorectal cancer: a Hellenic Cooperative Oncology Group phase III trial with collateral biomarker analysis

**DOI:** 10.1186/1471-2407-12-271

**Published:** 2012-06-29

**Authors:** Dimitrios Pectasides, George Papaxoinis, Konstantine T Kalogeras, Anastasia G Eleftheraki, Ioannis Xanthakis, Thomas Makatsoris, Epaminondas Samantas, Ioannis Varthalitis, Pavlos Papakostas, Nikitas Nikitas, Christos N Papandreou, George Pentheroudakis, Eleni Timotheadou, Angelos Koutras, Joseph Sgouros, Dimitrios Bafaloukos, George Klouvas, Theofanis Economopoulos, Konstantinos N Syrigos, George Fountzilas

**Affiliations:** 1 Oncology Section, Second Department of Internal Medicine, “Hippokration” Hospital, University of Athens School of Medicine, Athens, Greece; 2 Department of Medical Oncology, Papageorgiou Hospital, Aristotle University of Thessaloniki School of Medicine, Thessaloniki, Greece; 3 Translational Research Section, Hellenic Cooperative Oncology Group, Data Office, Athens, Greece; 4 Section of Biostatistics, Hellenic Cooperative Oncology Group, Data Office, Athens, Greece; 5 Division of Oncology, Department of Medicine, University Hospital of Patras, Rion, Greece; 6 Third Department of Medical Oncology, Agii Anargiri Cancer Hospital, Athens, Greece; 7 Oncology Department, General Hospital of Chania, Creta, Greece; 8 Department of Medical Oncology, Hippokration Hospital, Athens, Greece; 9 Department of Clinical Therapeutics, Alexandra Hospital, University of Athens School of Medicine, Athens, Greece; 10 Department of Internal Medicine, Oncology Section, Larissa University Hospital, Larissa, Greece; 11 Department of Medical Oncology, Ioannina University Hospital, Ioannina, Greece; 12 First Department of Medical Oncology, Metropolitan Hospital, Athens, Greece; 13 Second Department of Medical Oncology, Metropolitan Hospital, Athens, Greece; 14 Second Department of Internal Medicine, Propaedeutic, Oncology Section, Attikon University Hospital, Athens, Greece; 15 Oncology Unit, Third Department of Medicine, Athens Medical School, Sotiria General Hospital, Athens, Greece

**Keywords:** Angiogenic markers, Bevacizumab, Capecitabine, Chemotherapy, Colorectal cancer, Irinotecan

## Abstract

**Background:**

The aim was to compare two standard chemotherapy regimens combined with bevacizumab as first-line treatment in patients with metastatic colorectal cancer.

**Methods:**

Patients previously untreated for metastatic disease were randomized in: group A (irinotecan, capecitabine, bevacizumab, every 3 weeks; XELIRI-bevacizumab) and group B (irinotecan, leucovorin, fluorouracil, bevacizumab, every 2 weeks; FOLFIRI-bevacizumab). Primary endpoint was progression-free survival (PFS). Plasma concentrations of nitric oxide, osteopontin, TGF-β1 and VEGF-A were measured at baseline and during treatment.

**Results:**

Among 285 eligible patients, 143 were randomized to group A and 142 to group B. Fifty-five patients (38.5%) in group A and 57 (40.1%) in group B responded (p = 0.81). After a median follow-up of 42 months, median PFS was 10.2 and 10.8 months (p = 0.74), while median OS was 20.0 and 25.3 months (p = 0.099), for groups A and B, respectively. Most frequent grade 3–4 toxicities (group A vs group B) were neutropenia (13% vs 22%, p = 0.053) and diarrhea (19% vs 11%, p = 0.082). Baseline plasma osteopontin concentrations demonstrated prognostic significance for both PFS and OS.

**Conclusions:**

This trial did not show significant differences in efficacy between the groups. However, the toxicity profile was different. Baseline plasma osteopontin concentrations demonstrated independent prognostic significance. (Registration number: ACTRN12610000270011)

## Background

Colorectal cancer (CRC) is the third leading cause of cancer and cancer death in the US. Approximately, 15–20% of the patients have stage IV disease at diagnosis. Prognosis of this group of patients is poor, as only less than 10% of patients survive beyond 5 years. The introduction of new drugs in the systemic treatment of metastatic CRC (mCRC) during the last two decades has increased median survival in clinical trials from 6–9 months to beyond 2 years [1].

Irinotecan combined with 5-fluorouracil (5-FU)-based chemotherapy and bevacizumab (Bev) consist an established option in the treatment of mCRC [1]. Continuous infusion of 5-FU added to irinotecan (FOLFIRI) has been shown to be more effective and tolerable than bolus 5-FU [2]. However, this regimen requires hospitalization or the placement of central venous line. In contrast, the irinotecan-capecitabine combination (XELIRI) appears to be more convenient [2]. In the BICC-C randomized trial, XELIRI, compared with FOLFIRI, was associated with higher rates of serious nausea, vomiting, diarrhea, dehydration, hand-foot syndrome and treatment discontinuation. Also, progression-free survival was shorter in patients treated with XELIRI, when only patients, who completed treatment, were compared [2]. As a result, the XELIRI arm was discontinued before treatment amendment with the inclusion of Bev. Therefore, no comparison of FOLFIRI versus XELIRI combined with Bev exists in the literature.

On the other hand, although recent research has revealed a number of valuable predictive biomarkers of the efficacy of epidermal growth factor inhibitors, no such progress has been made with regard to the treatment with Bev [3].

The aim of the study was to compare the efficacy and toxicity of XELIRI vs FOLFIRI both combined with Bev in the treatment of mCRC. Also, angiogenic biomarkers were measured in the plasma of patients included in the study and were evaluated for their association with efficacy endpoints.

## Methods

### Patients

In this multicenter prospective randomized phase III trial, patients with stage IV CRC, previously untreated for metastatic disease, were enrolled. All patients had histologically or cytologically confirmed mCRC and two-dimensional measurable disease, with at least one lesion being ≥15 mm. Previous adjuvant or neoadjuvant chemotherapy should had been completed at least 4 months before enrollment and prior major surgery or radiotherapy 4 weeks before enrollment. Age was ≥18 years with performance status (PS) 0–2 (ECOG) and adequate bone marrow, renal and liver function.

The clinical protocol was approved by Institutional Review Boards (IRBs) in participating institutions and by the National Organization for Medicines. The trial was included in the Australian New Zealand Clinical Trials Registry (ANZCTR) and allocated the following Registration Number: ACTRN12610000270011. The translational research protocol was approved by the Bioethics Committee of the Aristotle University of Thessaloniki. All patients provided study specific written informed consent. In addition, patients who were willing to provide biological material for future translational research studies signed a separate informed consent.

### Treatment

Patients were randomly assigned to receive Bev 7.5 mg/Kg day 1, irinotecan 240 mg/m^2^ day 1 and capecitabine 1000 mg/m^2^ days 1–14 repeated every 21 days for 6 cycles (group A, XELIRI) or Bev 5 mg/Kg day 1, irinotecan 180 mg/m^2^ day 1, leucovorin 200 mg/m^2^ 2 h infusion day 1, 5-FU 400 mg/m^2^ IV bolus day 1 followed by a 5-FU 2,400 mg/m^2^ 46 h continuous infusion repeated every 14 days for 12 cycles (group B, FOLFIRI). Bev was not to be administered to patients who had a contraindication and optionally in elderly individuals who were more than 75 years old. During randomization, done centrally at the HeCOG Data Office, patients were stratified according to performance status (PS ECOG 0 vs 1–2), the presence or not of liver metastases and previous administration or not of adjuvant chemotherapy.

All adverse events were estimated according to the NCI CTC 3.0 grading scale. The next cycle was not administered unless the granulocyte number was ≥1,500/mm³, platelet number ≥100,000/mm³, and all non-hematological toxicities resolved to grade ≤1. In case of a 2-week delay, treatment could be interrupted, according to the investigator’s decision. Capecitabine was interrupted in case of hand-foot syndrome, mucositis and diarrhea grade 2, until toxicities resolved. The doses of capecitabine that had been omitted were not given at a later point. Administration of G-CSF and recombinant erythropoietin was allowed. Also, oral pyridoxine was allowed, administered as prophylaxis or the treatment of existing hand-foot syndrome.

### Evaluation of disease

Disease evaluation was carried out after 3 cycles of treatment in group A and after 6 cycles in group B, at the end of treatment, and every 3 months thereafter by chest X-rays and CT scans of the abdomen and pelvis. MRI or bone scan were allowed when indicated. Objective response classification was based on the RECIST 1.0 guidelines. No central review of the imaging material was done.

### Determination of circulating levels of plasma markers

Nine milliliters (mL) of blood were collected in EDTA tubes, in the morning (between 08.30 and 11.00 h), from 173 patients with mCRC before the initiation of the treatment (baseline sample) and from 51 patients during the antiangiogenic treatment with Bev (on-treatment sample, at least 3 weeks after treatment initiation). The plasma was separated by centrifugation (at 2,000 rpm) and aliquoted and frozen in −80 °C within 20 min from blood collection.

Plasma concentrations of nitric oxide (NO), osteopontin (OPN), transforming growth factor beta 1 (TGF-β1) and vascular endothelial growth factor A (VEGF-A) were measured, in duplicate, by solid-phase enzyme-linked immunosorbent (ELISA) assays using commercially available kits (VEGF-A from R&D Systems, Minneapolis, MN and the rest from Assay Designs, Ann Arbor, MI). Paired baseline samples and on-treatment samples from the same patient were run in the same ELISA plate for direct comparison. Due to the transient and volatile nature of NO, the assay used estimated NO blood levels by measuring its two stable breakdown products, nitrate (NO_3_) and nitrite (NO_2_), that can be easily detected by photometric means. The lower limit of detection was 3.13 μmol/L for the NO assay, 2.00 ng/mL for OPN, 31.20 pg/mL for TGF-β1, and 15.60 pg/mL for the VEGF-A assay. All assays were specific without significant cross-reactivity with other related molecules. The intra- and inter-assay coefficients of variation, established by the manufacturers for concentrations close to the center of the standard curves, were 3.1% and 4.2% for the NO assay, 3.7% and 9.2% for OPN, 6.1% and 12.3% for TGF-β1, and 4.5% and 7.0% for the VEGF-A assay, respectively.

### Statistical analysis

The primary endpoint of the study was to compare progression-free survival (PFS) between the two treatment schedules, while secondary endpoints were objective response rate (ORR), overall survival (OS) and the toxicity profile of the two groups of treatment, as well as the prognostic significance of the evaluated angiogenic markers. A sample of 300 patients was required for the study, to ensure an 80% power at the 5% level of significance, for a two-sided test of the hypothesis that a difference of ±12.5% in the PFS rate exists from a baseline PFS rate of 50%. Considering a 3% withdrawal rate, 309 patients needed to enter the study. The study was not terminated prematurely at the planned interim analysis, which was performed when 50% of the endpoints had been reached.

Pearson chi-square and Fisher’s exact tests were applied to compare patient characteristics, response and toxicity. The Wilcoxon signed rank test was used for comparison between baseline and on-treatment plasma concentrations. The Mann–Whitney test was used to evaluate differences in the distribution of baseline values or in the percent change (on-treatment sample value minus baseline value divided by the baseline value times 100) according to overall response.

PFS was calculated from the randomization date to the first progression of the disease or death from any cause. OS was calculated from the date of randomization to the date of death or last follow-up. The Kaplan-Meier method was used to estimate PFS, median follow-up and OS distributions, while the log-rank test was used to compare these distributions. PFS, OS and ORR were analyzed on an intent-to-treat basis, while in the safety analysis and the description of treatment characteristics only the treated population was included.

The median cut-off for all evaluated markers was used to dichotomize plasma concentrations into low and high. Cox proportional hazard regression analyses, adjusted for treatment, were performed for selected clinicopathological characteristics and plasma concentrations of markers to assess prognostic significance on OS and PFS.

Multivariate analysis was performed in the subpopulation of Bev treated patients. A backward selection procedure, based on the likelihood ratio statistic with a removal criterion p > 0.10, was used to determine the final model. This procedure begins by fitting a model with all the variables of interest and then, at each step, the variable showing the smallest contribution to the model is deleted until all the variables remaining in the model are significant at the p < 0.10 level. This procedure identified a subclass of significant variables among the following: treatment group (FOLFIRI vs XELIRI), age (≥60 vs <60), sex (women vs men), PS (1–2 vs 0), adjuvant chemotherapy (yes vs no), primary site (rectal vs colon), number of metastatic sites (≥3 vs 2 vs 1) and NO, OPN, TGF-β1 and VEGF-A plasma concentrations (high vs low). Data were analyzed using SPSS for Windows version 15 (SPSS Inc., Chicago, IL).

## Results

**Figure 1:**
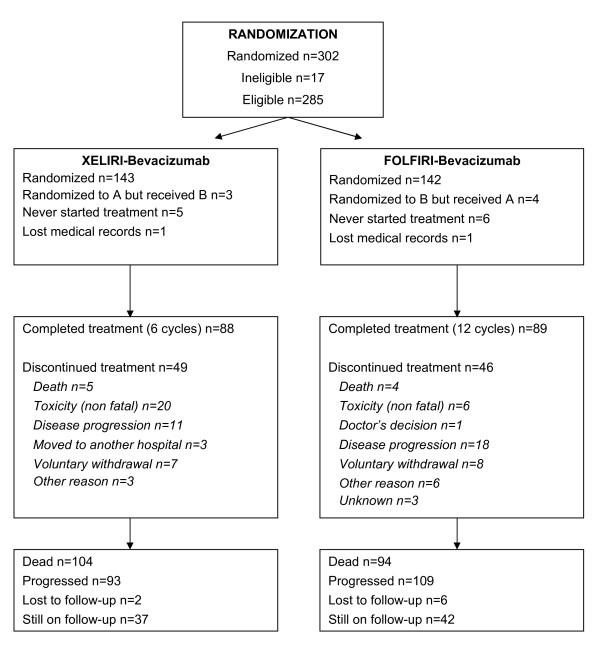
**Consort diagram: Among 285 eligible patients 143 were randomized in group A (XELIRI-Bev) and 142 in group B (FOLFIRI-Bev).** In group A 88 (61%) and in group B 89 (63%) of the patients completed treatment. Reasons of discontinuation were death, non-fatal toxicity, disease progression and doctor’s or patient’s decision. Discontinuation rates did not differ between the two groups (p = 0.80).

Between January 24, 2006, and January 21, 2008, 302 patients were enrolled in the study. Among them, 285 patients (94%) were eligible, 143 were randomized to group A and 142 to group B. The consort diagram for the patient population is illustrated in Figure 1. Table 1 shows the baseline characteristics of all eligible patients. There were no statistically significant differences in baseline characteristics between the two groups.

**Table 1:** Selected basic patient characteristics*

	**Group A**	**Group B**
	**(XELIRI-Bev)**	**(FOLFIRI-Bev)**
	**N = 143**	**N = 142**
**Age**		
Median (range)	66 (28–84)	66 (32–80)
	**N (%)**	**N (%)**
<60	40 (28)	44 (31)
≥60	103 (72)	98 (69)
**Gender**		
Male	79 (55)	92 (65)
Female	64 (45)	50 (35)
**PS (ECOG)**		
0	92 (64)	94 (66)
1	37 (26)	40 (28)
2	12 (8)	6 (4)
Missing data	2 (2)	2 (2)
**Primary location**		
Colon	98 (68)	85 (60)
Rectum	35 (25)	44 (31)
Rectosigmoid	3 (2)	8 (6)
Rectosigmoid and cecum	1 (1)	–
Misssing data	6 (4)	5 (3)
**Stage at diagnosis**		
I	2 (1)	3 (2)
II	5 (4)	11 (8)
III	23 (16)	26 (18)
IV	107 (75)	90 (63)
Missing data	6 (4)	12 (9)
**Previous adjuvant chemotherapy**		
No	114 (80)	112 (79)
Yes	27 (19)	27 (19)
Missing data	2 (1)	3 (2)
**Previous surgery**	114 (80)	123 (87)
**Symptomatic disease**		
Yes	70 (49)	66 (46)
No	56 (39)	54 (38)
Missing data	17 (12)	22 (16)
**Organs involved**		
Liver	103 (72)	101 (71)
Lung	51 (36)	39 (28)
Other	54 (38)	61 (43)
**Number of organs involved**		
1	84 (59)	85 (60)
2	39 (27)	43 (30)
≥3	19 (13)	14 (10)
Missing data	1 (1)	–

*Pearson chi-square and Fisher’s exact tests were applied to compare patient characteristics (all p values above 0.05).

### Treatment

Treatment and toxicity data are presented for the patients as treated, in contrast to the response and survival data and the consort diagram where the patient population is presented as randomized. In group A, 138 patients received 635 cycles of XELIRI (median, 6; range, 1–6) and in group B, 134 patients received 1,324 cycles of FOLFIRI (median, 12; range, 1–12). Among them, 89 patients (65%) completed treatment with XELIRI and 86 patients (64%) with FOLFIRI. In total, in group A, 42 patients (30%) and 50 patients (36%) required a dose reduction of irinotecan and capecitabine, respectively. In group B, 41 patients (31%) and 43 patients (32%) required a dose reduction of irinotecan and 5-FU, respectively. The number of cycles delivered at full dose was 472 (74%) in group A and 958 (72%) in group B. Median relative dose intensities in group A were 0.94 (range, 0.44–1.32) for irinotecan and 0.56 (range, 0.06–1.06) for capecitabine, while in group B dose intensities were 0.90 (range, 0.37–1.39) for irinotecan, 0.94 (range, 0.41–1.94) for bolus 5-FU and 0.90 (range, 0.31–1.08) for the 5-FU continuous infusion. Bev was administered in 237 (87%) of the patients (117 in group A and 120 in group B). The reasons of not administering Bev in 35 of the 272 treated patients were: a history of deep vein thrombosis (2), severe coronary artery disease (3), inadequately controlled arterial hypertension (13), other significant cardiac diseases (3), stroke (1), recent metastasectomy (1), brain metastasis with edema (1), advanced age (6), patient’s decision (1) and unknown reasons (4). In total, 64 patients in group A and 46 in group B received Bev as maintenance for a median of 4 cycles (range, 1–35) and 6 cycles (range, 2–48), respectively. Advanced age patients that did not receive Bev were balanced between groups: in group A, 3 out of 16 patients and in group B, 3 out of 17 patients aged >75 years did not receive Bev.

### Response

In group A, 55 patients (38.5%) achieved an objective response (complete response, 3.5%; partial response, 35%), 30 patients (21%) had stable disease and 15 (10.5%) progressive disease. In group B, 57 patients (40.1%) responded (complete response, 2.8%; partial response, 37.3%), 41 patients (28.9%) had stable disease and 16 (11.3%) progressive disease. Overall response rate did not differ significantly between the two groups (p = 0.81). In group A, 43 patients (30.1%) were not evaluated for response because of treatment discontinuation (24 patients, 16.8%), early death (5, 3.5%), missing data (3, 2.1%), or non-evaluable disease (11, 7.7%). In group B, 28 patients (19.7%) were not evaluated for response because of treatment discontinuation (5 patients, 3.5%), early death (2, 1.4%), or non-evaluable disease (21, 14.8%).

### Survival

**Figure 2:**
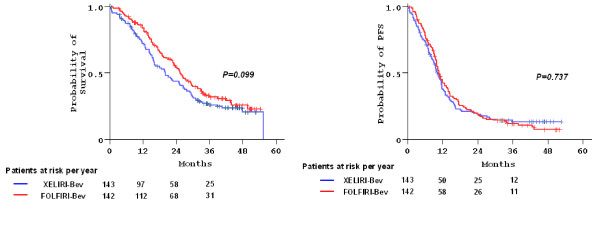
Kaplan-Meier curves for survival (left) and PFS (right) according to treatment allocation.

After a median follow-up of 42 months (range 0.4–55.4), 93 patients (65%) progressed and 104 (73%) died in group A, while 109 patients (77%) progressed and 94 (66%) died in group B. Median PFS was 10.2 months (95% confidence intervals [CI]: 9.0–11.5) in group A and 10.8 months (95% CI: 9.7–11.8) in group B (p = 0.74). Median OS was 20 months (95% CI: 15.4–24.6) in group A and 25.3 months (95% CI: 22.1–28.6) in group B (p = 0.099). Two-year PFS and OS rates were 18% and 44% in group A, and 17% and 54% in group B, respectively. Kaplan-Meier curves for OS and PFS according to treatment are shown in Figure 2.

### Toxicity

Grade 3–4 adverse events are shown in Table 2. The most common (group A vs group B) were neutropenia (13% vs 22%, Fisher’s exact test, p = 0.053) and diarrhea (19% vs 11%, Fisher’s exact test, p = 0.082). Vomiting was more frequent with XELIRI (5% vs 0%, Fisher’s exact test, p = 0.014). As for the Bev-treated patients most common serious toxicities were hypertension and thrombosis. Pulmonary embolism occurred only in one patient. No treatment-related deaths were recorded.

**Table 2:** Severe toxicity per treatment group (as treated population)*

	**Group A**				**Group B**			
	**(XELIRI-Bev)**				**(FOLFIRI-Bev)**			
	**N = 133**				**N = 132**			
	**Grade III**		**Grade IV**		**Grade III**		**Grade IV**	
	**N**	**%**	**N**	**%**	**N**	**%**	**N**	**%**
Anemia	–	–	–	–	1	1	1	1
Leucopenia	8	6	1	1	5	4	–	–
Neutropenia	11	8	6	5	25	19	4	3
Febrile Neutropenia	2	2	2	2	2	2	–	–
Thrombocytopenia	–	–	1	1	–	–	–	–
Infection	–	–	–	–	2	2	–	–
Fever	2	2	–	–	–	–	–	–
Fatigue	3	2	–	–	3	2	–	–
Anorexia	–	–	–	–	2	2	–	–
Nausea	1	1	–	–	–	–	–	–
Vomiting^**1**^	7	5	–	–	–	–	–	–
Mucositis	1	1	–	–	2	2	–	–
Diarrhea	24	18	1	1	14	11	–	–
Other gastrointestinal	–	–	–	–	1	1	–	–
Cholinergic syndrome	–	–	–	–	1	1	–	–
Hiccups	1	1	–	–	–	–	–	–
Voice changes	1	1	–	–	–	–	–	–
Transaminasemia	3	2	–	–	1	1	–	–
Hand–foot syndrome	1	1	–	–	1	1	–	–
Metabolic	3	2	1	1	4	3	2	2
Allergic reaction	1	1	–	–	–	–	–	–
Electrolyte disturbances	7	5	–	–	7	5	1	1
Hypertension	6	5	–	–	5	4	–	–
Thrombosis	4	3	–	–	5	4	2	2
Pulmonary embolism	–	–	–	–	–	–	1	1
Syncope	1	1	–	–	5	4	–	–
Cardiac	1	1	1	1	1	1	2	2
Hemorrhage	–	–	–	–	1	1	–	–

*Pearson chi-square and Fisher’s exact tests were applied to compare severe toxicities (all p values above 0.05, except for vomiting).

^**1**^Fisher’s exact test, p = 0.014.

### Translational research

Baseline plasma samples were collected from 200 patients. Among them, 59 also had an on-treatment plasma sample (at least 3 weeks after treatment initiation). Twenty-seven patients were excluded from the analysis because they did not receive Bev. Hence, 173 baseline and 51 on-treatment samples were evaluated in the analysis.

Plasma concentrations of all markers, including mean, median, minimum and maximun values, are shown in Table 3. Plasma levels of OPN, TGF-β1 and VEGF-A were significantly reduced during treatment (Wilcoxon’s test, p = 0.002, p = 0.025 and p < 0.001, respectively). Interestingly, this was not associated with response, except for TGF-β1, for which a significantly greater percent change was seen in responders versus non-responders (Mann–Whitney test, p = 0.012). It appears that treatment might had affected plasma concentrations of most of the markers, regardless of the type of response exhibited by the patients. In addition, baseline plasma levels of markers did not predict response.

**Table 3:** Descriptive characteristics for evaluated plasma markers

	**N**	**Mean**	**Median**	**SD**	**Min**	**Max**	**Wilcoxon’s p**
**NO (μmol/L)**							
Baseline samples	173	261.47	211.80	192.86	8.28	1,567.20	0.89
On-treatment samples*	51	237.09	227.10	140.68	42.69	606.70	
**OPN (ng/mL)**							
Baseline samples	173	98.68	62.56	89.29	23.12	512.00	0.002
On-treatment samples	51	75.42	54.72	76.32	25.44	507.52	
**TGF-β1(ng/mL)**							
Baseline samples	173	34.24	20.63	57.07	0.53	662.21	0.025
On-treatment samples	51	28.36	18.80	33.35	3.34	228.33	
**VEGF-A (pg/mL)**							
Baseline samples	172	122.54	57.85	190.33	15.60	1,601.31	<0.001
On-treatment samples	51	46.79	44.80	21.17	15.60	130.81	

*N*, number; *SD*, standard deviation; *Min*, minimum level detected; *Max*, maximum level detected.

*On-treatment samples, at least 3 weeks after treatment initiation.

**Figure 3:**
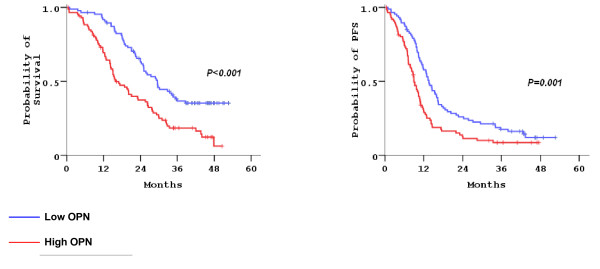
High baseline plasma OPN concentrations (above the median) were significantly associated with shorter OS and PFS.

In univariate analysis, the prognostic significance of increasing baseline plasma concentrations (used as a continuous variable) of most markers was prominent. Increasing baseline plasma concentrations of NO were significantly associated with shorter PFS (Hazard ration [HR] = 1.001, 95% CI: 1.001–1.002, p = 0.012) and OS (HR = 1.001, 95% CI: 1.001–1.002, p = 0.002). Also, increasing baseline plasma concentrations of OPN were associated with shorter PFS (HR = 1.006, 95% CI: 1.003–1.008, p < 0.001) and OS (HR = 1.008, 95% CI: 1.006–1.010, p < 0.001). Finally, increasing baseline plasma concentrations of VEGF-A were associated with adverse prognosis for OS (HR = 1.001, 95% CI: 1.000–1.002, p = 0.020). In addition, high baseline plasma OPN concentrations (above the median) were of adverse prognostic value for PFS (HR = 1.701, 95% CI: 1.229–2.354, p = 0.001) and OS (HR = 2.097, 95% CI: 1.465–3.000, p < 0.001), as shown in Figure 3. In multivariate analysis, as shown in Table 4, only high baseline plasma OPN concentrations demonstrated prognostic significance, for both PFS and OS, independently of treatment group, PS and number of organs involved. Similar results were observed when continuous values of plasma markers were included in the model.

**Table 4:** Multivariate Cox regression analysis (in the subpopulation of Bev-treated patients)*

	**Overall survival**			**Progression-free survival**		
	**HR**	**95**% **CI**	**Wald’s p**	**HR**	**95**% **CI**	**Wald’s p**
**Group**						
XELIRI-Bev	1			1		
FOLFIRI-Bev	0.842	0.587–1.207	0.35	0.949	0.682–1.321	0.76
**Performance status**						
0	1			1		
1–2	1.710	1.164–2.513	0.006	1.375	0.952–1.986	0.090
**Number of organs involved**						
1	1			1		
2	1.580	1.052–2.373	0.027	1.475	1.019–2.135	0.040
≥3	2.598	1.502–4.493	0.001	2.305	1.337–3.973	0.003
**Osteopontin (OPN)**						
Low	1			1		
High	1.632	1.116–2.387	0.012	1.467	1.044–2.060	0.027

*Both models remain the same whether continuous or binary variables of plasma concentrations of markers were used.

*HR*, hazard ratio; *CI*, confidence interval.

## Discussion

In the present randomized phase III trial, the primary endpoint, PFS, was comparable between the two treatment groups. Patients assigned to the XELIRI-Bev group had a median PFS of 10.2 months and those assigned to the FOLFIRI-Bev group 10.8 months. In contrast, in the BICC-C randomized trial [2], patients treated with FOLFIRI presented with a significantly longer PFS compared with those treated with XELIRI. Also, in the EORTC randomized trial [4], treatment with FOLFIRI resulted in a better PFS and OS than that with XELIRI, but the analysis included only 85 patients, due to the early termination of the trial and therefore no definitive conclusions could be made.

However, data regarding XELIRI combined with Bev are scarce in the literature. Most studies published in full paper form are retrospective in nature [5-7], with only Moehler et al. [8] recently reporting the results of a phase II trial. In the above studies, the major serious toxicities were diarrhea in 15–18% of the patients, nausea/vomiting in 3–7% and neutropenia in 6–7%. Doses were reduced in 31–62% of the patients, while treatment was interrupted due to toxicity in 11–18% of the patients. No treatment related deaths occurred. In the FNCLCC ACCORD 13/0503 randomized non-comparative study [9], the most frequent grade 3–4 events in patients treated with XELIRI-Bev were neutropenia (18%), asthenia (14%), diarrhea (12%), vomiting (7%) and hand-foot syndrome (6%). However, a lower than usual dose of irinotecan was administered (200 mg/m^2^).

In contrast, in the BICC-C trial [2], the incidence of the most prominent grade 3–4 adverse events with XELIRI was much higher: diarrhea (47%), neutropenia (32%), dehydration (19%), nausea (18%) and vomiting (16%). Twenty-five percent of the patients discontinued the regimen and the treatment was prematurely terminated. In the CAIRO trial [10], patients assigned to the XELIRI (combination) arm presented more frequently with diarrhea (27%), nausea-vomiting (9%), neutropenia (7%), febrile neutropenia (7%) and hand-foot syndrome (7%), as grade 3–4 toxicities. Also, in the EORTC 40015 study [4], patients treated with XELIRI demonstrated high rates of grade 3–4 toxicities: diarrhea (37%), neutropenia (14%), vomiting (7%) and nausea (4%). Fifty-three percent of the patients needed dose reduction and 14% succumbed from toxicity. It seems however, that more recent XELIRI studies present lower rates of serious toxicities. One possible explanation could be that there is now more experience with this regimen and patients are more efficiently instructed and managed. In the present phase III study, serious adverse events were relatively less frequent and only 15% of our patients discontinued treatment due to toxicity.

Among the above-mentioned studies with XELIRI-Bev, only a few [5,8,9] included previously untreated for metastatic disease CRC patients. Response rate was 35–67%, PFS was 12–13 months and OS 24 months. These results were slightly better than ours, but it should be mentioned that they are derived from phase II studies. The toxicity of the systemic treatment for CRC, in combination with the high cost, necessitates the discovery of predictive biomarkers of response.

Angiogenesis is necessary for cancer progression and constitutes a complex process with many different cooperating pathways, where VEGF-A, NO and TGF-β seem to have an important role. Early studies have demonstrated that immunohistochemical expression and high serum levels of VEGF-A and TGF-β1 are associated with adverse prognosis in CRC patients [11-17]. In contrast, the significance of NO blood levels in these patients is controversial [18,19]. In the present study, the first to evaluate plasma levels of the above molecules in Bev-treated mCRC patients, none of them was found to be associated with prognosis. However, in a recent study, baseline plasma levels of interleukin-8 were correlated with short PFS, while a number of other circulating angiogenic biomarkers, such as basic fibroblast growth factor, placental growth factor, hepatocyte growth factor, stromal-derived factor-1 and macrophage chemoattractant protein-3 were increased before the radiographic development of progressive disease in CRC patients treated with FOLFIRI-Bev [20].

Finally, OPN is a glycoprotein, which seems to induce cellular proliferation, survival, metastasis and angiogenesis [21]. More precisely, it is a soluble, secreted protein that can act as an autocrine and paracrine factor, as well as a modulator of cell adhesion through its interaction with integrins and CD44. It promotes multiple steps of cancer progression, such as cellular adhesion, proliferation, invasion, extracellular matrix degradation and metastasis, cancer immunotolerance and angiogenesis [21]. In CRC, OPN expression is proportionately increased with tumor stage and seems to be of adverse prognostic significance [22-26]. There are limited data in the literature with regard to circulating OPN in the blood of patients with colorectal cancer [27] and none, to our knowledge, with regard to a possible prognostic or predictive value of circulating OPN in CRC patients. In the present study, baseline plasma OPN concentrations were reduced with antiangiogenic treatment, while patients with low baseline plasma OPN levels had a significantly longer PFS and OS independently of other well-established prognostic factors, such as performance status and the number of organs involved. OPN measurements in plasma could therefore provide valuable prognostic information, which is robust, accurate and easily determined with a relatively low cost compared to other methods, which require adequate tumor tissue.

## Conclusions

In conclusion, in this trial no significant differences were demonstrated in efficacy between the two groups. However, the toxicity profile was different. Baseline plasma osteopontin concentrations demonstrated independent prognostic significance. Further studies are warranted to validate the prognostic value of OPN in independent cohorts of colorectal cancer patients.

## Competing Interest

The senior author (GF) has been on the Advisory Board of Pfizer.

The senior investigator (GF) has received Commercial Research Funding by Roche Hellas SA and Genesis Pharma SA, Athens, Greece.

## Authors’ contributions

Conception and design of the clinical study: DP, IX, TM, ES, IV, PP, NN, CNP, GP, ET, AK, JS, DF, GK, TE, KNS, GF. Conception and design of the translational research study: DP, GP, KTK, GF. Analysis and interpretation of data: DP, GP, KTK, AGE, GF. Drafted the manuscript: DP, GP, KTK. All authors read and approved the final manuscript.

## Pre-publication history

The pre-publication history for this paper can be accessed here:

http://www.biomedcentral.com/1471-2407/12/271/prepub
